# Chlamydia among Australian Aboriginal and/or Torres Strait Islander people attending sexual health services, general practices and Aboriginal community controlled health services

**DOI:** 10.1186/1472-6963-14-285

**Published:** 2014-07-01

**Authors:** James Ward, Jane Goller, Hammad Ali, Anna Bowring, Sophia Couzos, Mark Saunders, Phyllis Yau, John M Kaldor, Margaret Hellard, Rebecca J Guy, Basil Donovan

**Affiliations:** 1 Baker IDI Heart and Diabetes Institute, Alice Springs 0871 NT, Australia; 2 The Kirby Institute, The University of New South Wales, Sydney 2052 NSW, Australia; 3 The University of Melbourne, Grattan Street, Parkville VIC 3010, Australia; 4 James Cook University, James Cook Drive, Townsville QLD 4811, Australia; 5 National Aboriginal Community Controlled Health Organisation, Canberra City ACT 2601, Australia; 6 Sydney Sexual Health Centre, Sydney Hospital, Sydney 2000 NSW, Australia

**Keywords:** Chlamydia, Aboriginal and Torres Strait Islander people, Testing, Positivity, Indigenous, Australia

## Abstract

**Background:**

Chlamydia infections are notified at much higher rates in Aboriginal and/or Torres Strait Islander people compared to non-Indigenous people. The Australian Collaboration Chlamydia Enhanced Sentinel Surveillance System (ACCESS) was established to complement population-based surveillance.

**Methods:**

We describe patient demographics, completeness of recording of Aboriginal and/or Torres Strait Islander (‘Aboriginal’) status, chlamydia testing rates and positivity rates from the Aboriginal Community Controlled Health Service (ACCHSs), General Practice (GP) clinics and Sexual Health Services (SHSs) networks in ACCESS during 2009. Data were extracted from electronic medical records of each participating health service for consultations with patients aged 16–29 years and for chlamydia testing and positivity.

**Results:**

Data were included from 16–29 year olds attending six ACCHSs (n = 4,950); 22 SHSs (n = 20,691) and 25 GP clinics (n = 34,462). Aboriginal status was unknown for 79.3% of patients attending GP clinics, 4.5% attending SHSs and 3.8% of patients attending ACCHSs. Chlamydia testing rates among Aboriginal patients were 19.8% (95%CI:18.6%-21.0%) at ACCHSs, 75.5% (95% CI:72.5%-78.4%) at SHSs and 4.3% (95% CI: 2.6%-6.6%) at GP clinics. Positivity rates were highest in Aboriginal patients tested at SHSs at 22.7% (95% CI:19.5%-26.2%), followed by 15.8% (95% CI:3.8%-43.4%) at GP clinics and 8.6% at ACCHSs (95% CI:7.9%-12.4%). This compared with non-Indigenous patients positivity rates at SHSs of 12.7% (95% CI:12.2-13.2%); 8.6% (7.2%-11.3%) at GP clinics and 11.3% at ACCHSs (95% CI:15.4%-24.9%).

**Conclusions:**

Higher chlamydia positivity in Aboriginal people across a range of clinical services is reflected in national notification data. Targeted efforts are required to improve testing rates in primary care services; to improve identification of Aboriginal patients in mainstream services such as GP clinics; and to better engage with young Aboriginal Australians.

## Background

*Chlamydia trachomatis* is a sexually transmitted infection that is easily diagnosed and treated, but most infections go undetected since up to 80% are asymptomatic [1,2]. If left untreated chlamydia can cause pelvic inflammatory disease which in turn can lead to tubal factor infertility and ectopic pregnancy [3,4]. Chlamydia is the most commonly notified infection in Australia with more than 80,000 cases reported in 2013, a three-fold increase in the last decade [5]. The burden of chlamydia is highest among young Aboriginal and/or Torres Strait Islander people (hereafter ‘Aboriginal’) [6-8]. Notification rates among Aboriginal people are 24 times higher than among non-Indigenous people [9]; however, notification rates are subject to testing biases [10,11].

The focus of the Council of Australian Government’s commitment to *Closing the Gap* in health difference and life expectancy between non-Indigenous and Aboriginal peoples is principally directed towards mortality reduction from chronic disease and improvements to child and maternal health [12], with little focus on sexually transmissible infections (STIs) despite these causing substantial ill health among Aboriginal people. However, included within the *Closing the Gap* initiative are measures to improve surveillance systems that lead to increased understanding of disease among Aboriginal people and communities.

In this paper we report patient demographics, completeness of recording of patients’ Aboriginal status, chlamydia testing and positivity rates during 2009, within three ACCESS clinical networks providing primary care: sexual health services (SHSs), general practice (GP) clinics and Aboriginal Community Controlled Health Services (ACCHSs).

## Methods

### Study design

The Australian Collaboration for Chlamydia Enhanced Sentinel Surveillance System (ACCESS) program is an independent initiative that aims to complement and improve interpretation of passive chlamydia surveillance data among priority populations including Aboriginal people [13]. ACCESS aims to supplement notification data by gaining a better understanding of chlamydia testing and positivity rates within a range of health service networks.

The methods of the ACCESS systems have been described in detail elsewhere [13,14]. ACCESS includes five clinical networks made up of SHSs, family planning clinics, GP clinics, antenatal clinics and ACCHSs; and a laboratory network to collectively monitor the uptake and outcome of chlamydia testing in Australia using patient encounter data. ACCESS is managed by a coordinating committee and each of the six networks is overseen by a steering committee which manages all aspects of the project including development, implementation, interpretation and analysis of data from individual networks.

### Data inclusion

We included all consultation and routine chlamydia testing data from 25 GP clinics, 22 SHSs and six ACCHSs within ACCESS for both Aboriginal and non-Indigenous patients. These services nominated to participate in the ACCESS study, and were selected based on their size and geographical location to ensure national representatives [13]. A Memorandum of Understanding between research agencies and the National Aboriginal Community Controlled Health Organisation (NACCHO) supported ACCHSs involvement.

Data from GP clinics and ACCHSs were collected using software called GRHANITE developed by the Rural Clinical School at University of Melbourne [14]. GRHANITE collected client demographic, health service utilisation and testing and positivity data from each service’s electronic medical records. Data from the SHSs were directly extracted from their respective patient management systems. All extracted data was de-identified at the clinical service before sharing.

### Data analysis

Data on patient demographics, chlamydia testing and positivity were analysed for patients aged 16–29 years, who attended the services in 2009. We calculated: (i) the number of patients who were identified or identified as Aboriginal for each network; (ii) the proportion of patients tested at least once for chlamydia (testing rate) (iii) the proportion of those tested who had a positive test result (chlamydia positivity) where the result was known. All results were stratified by Aboriginal status, sex, age groups and clinic networks.

### Ethics

Ethical approval for the ACCHS network was gained from six Human Research Ethics Committees located at the Alfred Hospital, (Victoria); Aboriginal Health and Medical Research Council (New South Wales); Aboriginal Health Research Ethics Committee (South Australia); Central Australian Human Research Ethics Committee Northern Territory (NT)]; Western Australian (WA) Aboriginal Health Information and Ethics Committee; and the Human Research Ethics Committee of the NT Department of Health and Families and Menzies School of Health Research (NT). Approval for the GP network was granted by the Royal Australian College of General Practitioners (RACGP). Approval for the SHS network was granted by the Human Research Ethics Committees (HRECs) of St Vincent’s Hospital and the University of New South Wales with further ethical approval granted by the separate HRECs overlooking each of the 22 participating SHSs.

## Results

### Health services

Of the six ACCHS one was located in a metropolitan area and the remaining were in regional/remote areas, whereas the majority of the GP clinics (14 of 25; 56%) and SHSs (13 of 22; 59%) were located in metropolitan areas.

### Patient demographics

Within ACCHSs 4,950 unique patients aged 16–29 years attended during 2009. Of these, 60% were female, 33% were aged 16–19 years and 37% were aged 20–24 years. In the 25 GP clinics, 34,462 unique patients aged 16–29 years attended during 2009; 61% were female, 26% were aged 16–19 years and 38% aged 20–24 years. In the same year, 20,691 16–29 year olds attended the 22 SHSs, of these 48.9% were female; 17% were aged 16–19 years and 44% were aged 20–24 years (Table 1).

**Table 1:** Aboriginal status recording, by ACCESS network, age group, sex and area of residence, among 16–29 year olds

			**General practice clinics**^**(a)**^				**Sexual health services**^**(b)**^				**Aboriginal community controlled health services**^**(c)**^			
**Patients**			**Aboriginal**	**Non-indigenous**	**Not recorded**	**Patients**	**Aboriginal**	**Non-indigenous**	**Not recorded**	**Patients**	**Aboriginal**	**Non-indigenous**	**Not recorded**
			**n**	**n (%)**	**n (%)**	**n (%)**	**n**	**n (%)**	**n (%)**	**n (%)**	**n**	**n (%)**	**n (%)**	**n (%)**
Males	Age group (years)	16-19	3,604	50 (1.4)	1,164 (32.3)	2,390 (66.3)	1379	148 (10.7)	1202 (87.2)	29 (2.1)	657	582 (88.6)	58 (8.8)	17 (2.6)
		20-24	4,920	53 (1.1)	1,461 (29.7)	3,406 (69.2)	4716	128 (2.7)	4404 (93.4)	184 (3.9)	683	575 (84.2)	77 (11.3)	31 (4.5)
		25-29	4,751	37 (0.8)	1,469 (30.9)	3,245 (68.2)	4484	58 (1.3)	4219 (94.1)	207 (4.6)	624	512 (82.1)	76 (12.2)	36 (5.8)
	Area of residence	Metro	7,850	113 (1.4)	2,261 (28.8)	5,475 (69.7)	6990	78 (1.1)	6612 (94.6)	300 (4.3)	403	332 (82.4)	63 (15.6)	8 (2.0)
		Regional/ remote	5,324	27 (0.5)	1,813 (34.0)	3,485 (65.5)	2669	231 (8.6)	2377 (89.1)	61 (2.3)	1528	1318 (86.3)	136 (8.9)	74 (4.8)
Female	Age group (years)	16-19	5,410	98 (1.8)	1,822 (33.7)	3,490 (64.5)	2233	266 (11.9)	1910 (85.5)	57 (2.6)	962	844 (87.7)	89 (9.3)	29 (3.0)
		20-24	8,059	112 (1.4)	2,510 (31.1)	5,437 (67.5)	4380	140 (3.2)	4028 (92.0)	212 (4.8)	1,126	940 (83.5)	140 (12.4)	46 (4.1)
		25-29	7,718	97 (1.3)	2,333 (30.2)	5,288 (68.5)	3499	102 (2.9)	3165 (90.5)	232 (6.6)	898	754 (84.0)	116 (12.9)	28 (3.1)
	Area of residence	Metro	12,051	228 (1.9)	3,337 (27.7)	8,486 (70.4)	6076	93 (1.5)	5633 (92.7)	350 (5.8)	571	492 (86.2)	74 (13.0)	5 (0.9)
		Regional/ remote	8,970	74 (0.8)	3,295 (36.7)	5,601 (62.4)	3141	363 (11.6)	2699 (85.9)	79 (2.5)	2380	2023 (85.0)	261 (11.0)	96 (4.0)

^(a)^based on 25 clinics.

^(b)^based on 22 clinics.

^(c)^based on 6 clinics.

### Aboriginal status

The majority of patients (85%) attending ACCHSs were identified as Aboriginal, 11% as non-Indigenous, and in 4% this status was not recorded. Of all patients attending GP clinics, 1.3% were recorded as being Aboriginal, 31.2% as non-Indigenous, and for 67.5% of patients Aboriginal status was not recorded. Aboriginal patients comprised, 4.1% of the total patients seen at SHSs, with 91.5% recorded as non-Indigenous and for 4.3% Aboriginal status was not recorded (Table 1).

### Chlamydia testing rates

Chlamydia testing rates in each of the networks among Aboriginal patients were: 19.8% (95% CI:18.6%-21.0%) attending ACCHSs; 4.3% (95% CI:2.6-6.6%) at GP clinics, and 75.5% (95% CI:72.5%-78.4%) at SHSs (p < 0.01) (Table 2). Whereas, in non-Indigenous people, the testing rates were: 8.6% (95% CI:8.0%-9.1%) at ACCHSs, 11.2% (95% CI:8.6%-14.1%) at GP clinics, and 78.3% (95% CI:77.7%-78.9%) at SHSs (p < 0.01). Testing rates were higher in both Aboriginal and non-Indigenous males attending SHSs, 81.7% vs. 79.9% respectively; compared to 71.5% of females (both Aboriginal and non-Indigenous) (p < 0.01 for both). Conversely testing rates were higher for females both Aboriginal and non-Indigenous at ACCHS compared to males (22.5% vs 15.6%, p < 0.01 and 13.7% vs. 7.6%, respectively (p < 0.05 for both). Testing rates were similar between Aboriginal and non-Indigenous patients, both males and females at GP clinics (5.2% vs 2.1%; p = 0.136).

**Table 2:** Chlamydia testing and positivity rate, by Aboriginal status, ACCESS network, age group, sex and area of residence, among 16–29 year olds

**Aboriginal**	**Category**	**Breakdown**	**General practice clinics (a)**			**Sexual health services (b)**			**Aboriginal community controlled health services (c)**		
			**Patients**	**Tested**	Positive*	**Patients**	**Tested**	**Positive**	**Patients**	**Tested**	Positive*
			**N**	**n (%)**	**n (%)**	**n**	**n (%)**	**n (%)**	**n**	**n (%)**	**n (%)**
**Aboriginal and/or Torres Strait Islander**	All		447	19 (4.3)	3 (15.8)	845	638 (75.5)	145 (22.7)	4207	833 (19.8)	72 (8.6)
	Sex	Male	140	3 (2.1)	1 (33.3)	334	273 (81.7)	67 (24.5)	1669	261 (15.6)	18 (8.1)
		Female	307	16 (5.2)	2 (13.3)	508	363 (71.5)	78 (21.5)	2538	572 (22.5)	54 (9.4)
	Age group (years)	16-19	148	7 (4.7)	2 (28.6)	415	321 (77.3)	76 (23.7)	1426	268 (18.8)	27 (10.1)
		20-24	165	7 (4.2)	1 (14.3)	269	208 (77.3)	52 (25.0)	1515	308 (20.3)	29 (9.4)
		25-29	134	5 (3.7)	-(0.0)	161	109 (67.7)	17 (15.6)	1266	257 (20.3)	16 (6.2)
	Patient area of residence	Metropolitan	341	8 (2.3)	-(0.0)	172	115 (66.9)	17 (14.8)	824	171 (20.8)	14 (8.2)
		Regional/remote	101	11 (10.9)	3 (27.3)	596	468 (78.5)	119 (25.4)	3341	659 (19.7)	57 (8.6)
**Non-Indigenous**	All		10,759	920 (8.6)	75 (8.2)	18,968	14851 (78.3)	1887 (12.7)	556	62 (11.2)	7 (11.3)
	Sex	Male	4,094	218 (5.3)	22 (10.1)	9,825	7848 (79.9)	1051 (13.4)	211	16 (7.6)	2 (12.5)
		Female	6,665	702 (10.5)	53 (7.5)	9,103	6979 (76.7)	833 (11.9)	345	46 (13.3)	5 (10.9)
	Age group (years)	16-19	2,986	238 (8.0)	25 (10.5)	3,116	2414 (77.5)	373 (15.5)	147	12 (8.2)	2 (16.7)
		20-24	3,971	409 (10.3)	42 (10.4)	8,444	6693 (79.3)	915 (13.7)	217	29 (13.2)	4 (13.8)
		25-29	3,802	273 (7.2)	8 (2.9)	7,408	5744 (77.5)	599 (10.4)	192	21 (10.9)	1 (4.8)
	Patient area of residence	Metropolitan	5,598	448 (8.0)	28 (6.3)	12,207	9580 (78.0)	1124 (11.7)	137	8 (5.8)	2 (25.0)
		Regional/remote	5,108	470 (9.2)	47 (10.0)	5,082	3899 (76.7)	587 (15.1)	397	53 (13.4)	5 (9.4)

a) based on 25 clinics b) based on 22 clinics c) based on 6 clinics.

*Positivity rates based only on individuals tested for whom a result was available.

### Chlamydia positivity rates

Among Aboriginal patients, chlamydia positivity at ACCHSs was 8.6% (95% CI:6.8%-10.8%) (72 of 833 patients tested), 15.8% (95% CI:3.4%-39.6%) (3/19) at GP clinics, and 22.7% (95% CI:19.5%-26.2%) (145/638) at SHSs (p < 0.01). Among non-Indigenous people, chlamydia positivity at ACCHS was 11.3% (95% CI:4.7%-22.0%) (7/62), 8.2% (95% CI:6.4%-10.1%) (75/920) at GP clinics, and 12.7% (95% CI:12.2%-13.2%) (1887/14851) at SHSs (p < 0.01) (Table 2).

Among Aboriginal females, chlamydia positivity was highest at SHSs; 21.5% (95% CI:17.3%-26.1%), while those tested at ACCHSs had a positivity rate of 9.4% (95% CI:7.2%-12.1%,p < 0.01). Among Aboriginal males chlamydia positivity rates within SHSs was 24.5% (95% CI:19.5%-30.0%) and within ACCHSs 8.1% (95% CI:4.8%-12.5%,p < 0.01) (Table 2).

## Discussion

This report adds significantly to our understanding of the health care utilisation by young Aboriginal people and complements STI notification data by informing chlamydia positivity across a range of primary care services- two generalist (GP and ACCHSs) and one specialised (SHSs). The study is the first comprehensive analyses, and certainly the biggest that compares Aboriginal/non-Aboriginal populations using the same recruitment methods for both populations. Previous comparisons have been based virtually entirely on notification data, which is subject to variabililty due to dependence on testing uptake.

Data from three different types of primary care services are represented, covering a period of 12 months with over 60,100 patients. Elsewhere, we reported the representativeness of participating sites according to Australian population distributions [13]. Accordingly, results particularly related to Chlamydia testing and positivity are likely to be generalisable to the Australian population aged 16–29 years.

At the participating services, 4.1% of clients at SHSs were identified as Aboriginal, compared to 1.3% at GP clinics and 85% at ACCHSs. Aboriginal attendances in GPs and ACCHSs from this dataset were consistent with those reported in national audits across all ages, and affirm that young Aboriginal people only make up a small proportion of those attending mainstream encounters the patient identified as sexual health services for STI care and management. Of all patients attending GP clinics in 2011–2012 1.5% were identified as Aboriginal [15] while 78% of patients who attended ACCHS in 2010–2011 were identified as Aboriginal [16]. Given that young Aboriginal people make up 4% of Australia’s total 15–29 year old population [17], further efforts are required to make GP services more accessible to them. It should be noted however that young men accessed SHSs more than the GP clinics and ACCHSs; this is likely for two reasons (i) either they are accessing clinics on their own or (ii) they attend as named contacts. Whatever the case, all services should be encouraged to promote their services to young men.

The Aboriginal status of patients was better recorded at ACCHSs and SHSs compared with GP clinics where only one third of patients status was recorded. Improving data quality on Aboriginal status within GP clinics is a priority of Australian Governments [18] with the Close the Gap Indigenous health incentive payment aiming to improve recording of Aboriginal status [19].

This study confirms that the majority of ACCHSs patients are Aboriginal people and that people aged 16–29 years attend these services for health care, including for STI screening. This concurs with two previous studies, the first, a cross sectional survey of young people aged 16–29 years where 54% reported that they had an STI test in the previous year and did so at an ACCHS [20], and a second study of resilience related to STI and blood borne viruses whereby young people reported positive aspects of attending an ACCHS for STI and BBV screening and management. In the latter study, young Aboriginal people described the comfort and understanding they experienced at the ACCHS; and positive personal relationships especially with Indigenous care providers [21]. Our results support the central role that ACCHSs play in STI service provision for Aboriginal people.

Further efforts are required to improve STI testing rates in primary care. Testing rates among Aboriginal patients attending ACCHSs and GP clinics were quite different with testing rates within ACCHS almost five times greater than that offered by GPs for both Aboriginal and non-Indigenous patients. As expected, testing rates were high among both Aboriginal and non-Indigenous patients attending SHSs. There are two main reasons to test for Chlamydia- to screen those who are asymptomatic or to diagnose symptomatic infection. Thus, chlamydia testing rates of services may reflect their adherence to screening protocols, as well as the burden of disease affecting their populations. With regards to screening, clinical practice guidelines recommend that all sexually active people up to the age of 29 years be offered a chlamydia test at least annually [22,23]. The lower rates of testing within GPs and ACCHSs compared with SHSs could be due to a number of factors: patients declining testing; patients not being offered testing (staff lacking awareness of screening guidelines, due lack of time and awareness about chlamydia testing amongst clinic staff, or sensitivities regarding sexual health issues); or other clinical priorities [24-26]. In order to achieve higher chlamydia testing rates in GP and ACCHS settings, supportive quality improvement strategies are warranted [27,28] as well as initiatives such as offering a test to all patients prior to the consultation [29] Quality improvement audits are a key feature of the *Closing the Gap* funding initiatives particularly involving ACCHSs [30] but do not currently explore STI testing patterns.

Mathematical modelling has suggested that increasing rates of chlamydia testing to 40% in males and females aged less than 25 years or to 20% in young people aged less than 30 years could halve the prevalence of chlamydia within ten years; with most of this decline occurring within the first four years [31]. If the intent is to achieve high chlamydia testing rates in the population, health promotion and clinical education and systems within ACCHS and GPs should address the different health care utilisation patterns of Aboriginal females and males; since in most ACCESS sites a higher proportion of females either attended or were tested than males.

A key strength of this study is that the results reflect actual clinical practice in the participating health services. All data were collected retrospectively and thus did not influence the decision to offer a chlamydia test or not. In addition, within each network, study data were collected from services that are geographically dispersed across the country; thus reducing biases introduced when data are reported from one service or region.

A limitation of the study was the poor identification of Aboriginal patients at mainstream GP clinics with more than two thirds of patients having an unknown Aboriginal status. The poor completeness of Aboriginal status in GP clinics means we cannot be confident about the true testing and positivity rates in Aboriginal patients. In addition, the study did not record patients who declined to have a test when offered one. Clinical audits would be needed to measure the extent of decline in any of these primary care facilities. Further, we were not able to identify symptomatic patients from asymptomatic patients making interpretation of positivity results difficult to correlate with community prevalence rates. Only first patient visits involving Chlamydia testing were counted, thus excluding subsequent testing and positivity data. Finally, in view of the small sample size of GP clinics and ACCHSs in the ACCESS network, these findings may not be representative of all such health services because of the small sample size of these clinics in Australia.

## Conclusions

In conclusion, this study provides a snapshot of chlamydia testing and positivity among young people attending three types of primary care health services. Improved completeness of recording of Aboriginal status should be a priority in GP clinics, not only to evaluate chlamydia testing and prevention programs but also for other *Closing the Gap* initiatives that are directed at mainstream general practice [32]. Quality improvement initiatives will play a key role in influencing adherence to clinical practice guideline recommendations for chlamydia testing and are becoming a feature of current funding programs. More research is needed to determine how effective mainstream strategies are at engaging young Aboriginal people to the range of health services available to them.

## Competing interests

The authors declare that they have no competing interests.

## Authors’ contributions

JW conceptualized the study. JW, JG, HA and AB conducted the analysis. JW drafted the manuscript with assistance from JG, HA, RJG, SC and BD. JW, JMK, MH, RJG and BD are investigators on ACCESS. All authors contributed to the manuscript and read and approved the final version.

## Pre-publication history

The pre-publication history for this paper can be accessed here:

http://www.biomedcentral.com/1472-6963/14/285/prepub
